# Prevalence of depressive symptoms and factors associated with it in type 2 diabetic patients: a cross-sectional study in China

**DOI:** 10.1186/s12889-015-1567-y

**Published:** 2015-02-25

**Authors:** Linchuang Wang, Rui Song, Zhigang Chen, Jun Wang, Feng Ling

**Affiliations:** Longhua Street Community Health Center, Xuhui Shanghai, China; Center for Disease Control and Prevention of Xuhui, Shanghai, China

**Keywords:** Type 2 diabetes mellitus, Depressive symptoms, Risk factors

## Abstract

**Background:**

Depressive symptoms in patients with type 2 diabetes mellitus (T2DM) have attracted much attention in recent years, and negatively affect the health of diabetic patients in numerous ways. This study evaluated the prevalence rate of depressive symptoms in T2DM patients in Shanghai, and the potential factors that may be associated with depressive symptoms in this select population.

**Methods:**

A total of 865 T2DM patients were recruited from Longhua Street, Xuhui, Shanghai by simple random sampling, and all the patients were assessed with the Zung Self-rating Depression Scale to screen for depressive symptoms. Factors associated with depressive symptoms were analyzed by logistic regression.

**Results:**

Among the 865 patients (403 were male, 462 were female, average age 70.13 ± 20.33 years), 304 (35.1%) patients were categorized as having depressive symptoms. Rates of myocardial infarction and stress in one month were higher in the depressive symptoms group than in the non-depressive symptoms group by the *X*^*2*^ test. Rates of patients having a job, having a college education or above, and sleeping less than 7 h/24 h day were also higher in the depressive symptoms group by the *X*^*2*^ test. Body mass index, and levels of total cholesterol, triglyceride, urea, creatinine, uric acid, and homocysteine were higher in the depressive symptoms group by the independent samples t test and non-parametric test. Sleeping hours, history of myocardial infarction, stress in one month, working status, and total cholesterol were significantly associated with depressive symptoms (p < 0.05).

**Conclusions:**

In the Chinese population analyzed in this study, the prevalence rate of depressive symptoms in patients with T2DM was high. Further research on the relationship between diabetes and depressive symptoms is necessary in a wider Chinese population.

## Background

Type 2 diabetes mellitus (T2DM) is one of the most common chronic diseases worldwide, and its prevalence rate has been rising each year. It is anticipated that the global prevalence of T2DM will increase to 438 million by the year 2030 [1]. People with diabetes have a mortality rate that is twice as high as people of a similar age without diabetes. In recent years, it was found that people with diabetes are at an increased risk of experiencing depressive conditions in their lifetime. For example, individuals with diabetes mellitus have been shown to have twice the risk of depression as those without diabetes [2], a finding that has been supported by other studies [3]. Importantly, diabetic patients with depression were shown to have a significantly higher rate of negative outcomes such as diabetic complications [4], cardiovascular disease, and all-cause mortality risk [5] than those without depression, and individuals with depression were less likely to adhere to their medications than those without depression [6,7]. In addition, in comparisons of patients with T2DM and depression with those without depression, the risk of dementia was significantly increased in patients having by both T2DM and depression [8]. Numerous studies have reported that the coexistence of diabetes and depression was associated with increased healthcare costs [9,10]. It was also noted that effective treatment for depression may greatly attenuate the strong link between depression and mortality in an older population with T2DM [11]. Sadly, this depression often remains unrecognized and untreated [12], thus increasing the prevalence of depression among people with T2DM [12,13]. Therefore, it is critical to diagnose depression in diabetic patients as early as possible and provide them with the corresponding treatments.

In Beijing, a city in northern China and the capital of the country, it was found that the prevalence rate of depressive symptoms in T2DM patients was 44.23% [14]. However, studies on the prevalence of depressive symptoms in patients with T2DM in Shanghai (a city in eastern China and the biggest city in country) were rare. In view of the high prevalence and the marked differences reported within China, it would also be important to learn about the prevalence of depressive symptoms in T2DM patients in Shanghai.

The purpose of this study was to identify the prevalence rate of depressive symptoms in T2DM patients and the risk factors related to depressive symptoms in Shanghai. These consequences need to be urgently considered to find efficient intervention methods to reduce the occurrence of complications and deaths of patients with both T2DM and depressive symptoms.

## Methods

### Research sample

The patients surveyed in this cross-sectional study were selected randomly from all the T2DM patients in Longhua Street, Xuhui, Shanghai. The calculation of the sample size was based on the finding that the prevalence rate of depressive symptoms in T2DM patients was 35.6% in the center region of China; the level of α = 0.05, the allowable error = 10%, and invalid questionnaire rate = 10%. Based on these facts it was calculated that a total of 796 cases were needed for the present study. We were able to collect a total of 910 cases. Questionnaires with responses that did not meet the inclusion criteria and lacked sufficient data were excluded. Finally, 865 participants were included in this study. Subjects who met the following inclusion criteria were eligible for participation: (1) diagnosed as T2DM according to World Health Organization criteria in 1999; (2) without mental disease and related family history; (3) no history of drugs that influenced mental health; and (4) individuals who volunteered to participate in this study. Individuals who experienced acute complications of diabetes, those who indicated that they had serious heart, lung, kidney, or other organ dysfunctions, or had severe infection were excluded from the study.

The research protocol was approved by the Ethics Committee of Longhua Street Community Health Center. Researchers explained the purpose of the study and procedures to the participants before their interviews. All the participants were informed that their participation was voluntary, and each of the participants signed an informed consent form.

### Measurements and assessments of depressive status

Demographic and clinical characteristics of the patients, such as age, sex, body mass index (BMI), work status, fasting blood glucose, triglycerides (TG), total cholesterol (TC), pre-existing medical conditions, and history of stress in one month, were collected in this study. Among them, “history of stress in one month” meant that the patients experienced stressful events such as changes in their work status or lost their jobs, or their lineal relatives got sick or died within a one month timeframe.

The Chinese version of the Zung Self-rating Depression Scale (ZSDS) was used to screen depressive symptoms. ZSDS was identified as a useful and well-validated questionnaire by Chinese psychiatrists [15]. ZSDS contains 20 items. Every item can be scored from 1 (where depressive symptoms are very seldom) to 4 (where depressive symptoms are most of the time). The total score was defined as the sum of the total numbers obtained in the 20 items, and the standardized score is equal to 1.25 times the total score. Subjects were classified according to their standardized scores. According to the Chinese norm of ZSDS, depressive symptoms were defined as a standardized score of 53 or higher; 53–62 indicates mild depressive symptoms, 63–72 indicates moderate depressive symptoms, and >72 indicates severe depressive symptoms [16]. In this study, the patients were divided into two groups for analysis: patients with their standardized scores ≥53 were classified as the “depressive symptoms” group, and patients with their standardized <53 were classified as the “non-depressive symptoms” group.

### Statistical analyses

Statistical analyses were performed with SPSS 19.0. Continuous variables were expressed as mean ± standard deviation. Discrete variables were expressed as absolute values and percentages. The comparison of continuous variables used the independent sample t-test or non-parametric test. Pearson’s chi-square test or Fisher’s exact test was used for the comparison of rates of discrete variables. Logistic regression analysis was used to evaluate the variables correlated with depressive symptoms in diabetic patients. p < 0.05 was considered as statistically significant.

## Results

### Demographic characteristics and disease-related parameters of diabetic patients in the depressive symptoms group and the non-depressive symptoms group

A total of 865 patients were included in the present study, and the response rate was 95.1%. Among them, 403 (46.6%) were men and 462 (53.4%) were women. The mean (± standard deviation) age of the participants was 70.13 (±20.33) years. In this study, the prevalence rate of depressive symptoms was 35.1%, and the rates of mild, moderate, and severe depressive symptoms were 28.2%, 6.7%, and 0.2%, respectively.

The participants’ main demographic characteristics are shown in Table 1. The rates of patients with a job and patients with a college education or above were higher in the depressive symptoms group than in the non-depressive symptoms group, and the average salary was higher in the depressive symptoms group.

**Table 1:** **Patient demographic characteristics**

**Characteristic**	**Non-depressive symptoms group**	**Depressive symptoms group**	**Overall subjects**	***P*** **-value**
	**(ZSDS score <53)**	**(ZSDS score ≥ 53)**		
Gender (male,n/%)	263/46.9	140/46.1	403/46.6	0.816
Age (years)	69.25 ± 18.51	71.76 ± 23.26	70.13 ± 20.33	0.083
Marriage status (married, n/%)	495/88.7	263/87.7	758/88.3	0.65
Working status (on job, n/%)	216/38.5	146/48.0	362/41.8	0.007
Educational background (college and above, n/%)	98/17.5	76/25.6	174/20.3	0.005
Average salary (RMB)	4022.47 ± 1726.62	4878.87 ± 1909.81	4254.18 ± 1817.02	0.000

The participants’ disease-related parameters are presented in Table 2. Diabetic patients with depressive symptoms had higher rates of myocardial infarction and stress in one month, and the rate of participants sleeping more than 7 h/24 h day was lower in the depressive symptoms group. BMI and the levels of TC, TG, urea, creatinine, uric acid, and homocysteine were higher in the depressive symptoms group than in the non-depressive symptoms group.

**Table 2:** **Disease-related parameters of T2DM patients with and without depressive symptoms**

**Characteristic**	**Non-depressive symptoms group**	**Depressive symptoms group**	**Overall subjects**	***P*** **-value**
	**(ZSDS score <53)**	**(ZSDS score ≥ 53)**		
History(n/%)				
Hypertension	424/75.6	240/79.2	664/76.9	0.23
Valvular heart disease	7/1.3	5/1.7	12/1.4	0.63
Dyslipidaemia	197/35.2	104/34.3	301/34.9	0.801
Myocardial infarction	7/1.2	11/3.6	18/2.1	0.020
Stress in one month	4/0.7	9/3.0	13/1.5	0.01
Sleeping hours(>7 h / 24 h day, n/%)	148/26.6	42/16.3	190/23.3	0.001
BMI (kg/m^2^)	24.10 ± 3.11	24.54 ± 3.23	24.25 ± 3.16	0.047
SBP (mmHg)	127.84 ± 9.68	127.34 ± 9.95	127.66 ± 9.77	0.47
DBP (mmHg)	77.61 ± 6.10	77.40 ± 6.44	77.53 ± 6.22	0.64
FBG (mmol/L)	7.05 ± 1.73	6.94 ± 1.56	7.01 ± 1.67	0.39
HbA1C (%)	6.69 ± 1.31	6.62 ± 0.93	6.67 ± 1.19	0.40
TC (mmol/L)	5.23 ± 1.09	5.82 ± 2.51	5.23 ± 1.09	0.00
TG (mmol/L)	1.96 ± 1.13	2.13 ± 1.10	2.02 ± 1.12	0.036
Urea (mmol/L)	6.71 ± 4.00	15.08 ± 22.11	10.06 ± 14.88	0.00
Creatinine (μmol/L)	72.40 ± 25.12	80.15 ± 24.92	75.58 ± 25.31	0.00
Uric acid (μmol/L)	294.49 ± 89.86	308.55 ± 77.81	300.25 ± 85.35	0.038
Homocysteine (μmol/L)	8.53 ± 4.47	10.93 ± 9.44	9.49 ± 6.98	0.00
Diabetes duration (years)	8.69 ± 6.89	9.58 ± 7.32	9.00 ± 7.05	0.077

### Factors associated with depressive symptoms among participants with diabetes by logistic regression

Table 3 shows factors associated with depressive symptoms among participants with diabetes. Participants who slept less than 7 h/24 h day had an average of 2.52 (95% confidence interval [CI] 1.59–3.98) higher depressive symptoms incidence compared with those who slept more than 7 h/24 h day (p < 0.001). Participants with myocardial infarction had an average of 3.91 (95% CI 1.16–13.22) higher depressive symptoms incidence compared with those without myocardial infarction (p = 0.028). Participants who had a job had an average of 1.68 (95% CI 1.13–2.48) higher depressive symptoms incidence compared with those who did not have a job (p = 0.010). Participants who had a history of stress in one month had an average of 4.49 (95% CI 1.08–18.62) higher depressive symptoms incidence compared with those without stress (p = 0.039). When TC raised 1 mmol/L, participants had an average of 1.29 (95% CI 1.09-1.53) higher depressive symptoms incidence (p = 0.003)

**Table 3:** **Risk factors associated with depressive symptoms in diabetic patients by logistic regression analysis**

**Characteristic**	***P*** **-value**	**OR**	**95% CI**
Sleeping hours (reference > =7 h/24 h day)	0.000	2.52	1.59-3.98
History of myocardial infarction(reference no MI)	0.028	3.91	1.16-13.22
Working status (reference not on job)	0.010	1.68	1.13-2.48
History of stress in one month (reference no stress)	0.039	4.49	1.08-18.62
TC (mmol/L)	0.003	1.29	1.09-1.53

## Discussion

This study shows for the first time the prevalence of depressive symptoms in a large group of patients with T2DM in Shanghai. Importantly, the study was conducted at the community level and the selection bias was reduced.

In this community-based sample of adult patients with T2DM, it was found that the prevalence rate of depressive symptoms in the participants was 35.1%. This rate was different from and lower than the rate in Beijing [14]. This may be because of the possibility that people in different areas of China have different living habits, and thus more research in this field may be needed with the inclusion of more cities in China. Although the rate of depressive symptoms in Shanghai was not as high as rates of other cities in China, none of the patients was aware of his/her depressive symptoms, and therefore his/her depression did not get timely treatment. Evidence shows that efficacious pharmacological and psychosocial treatments are available to patients with depressive symptoms [17,18], but if a patient declined medication therapy, the physician could recommend interpersonal psychotherapy from a healthcare manager. If diabetic patients with depressive symptoms were treated in a timely manner, it may reduce the occurrence of complications and even death.

The second goal of this study was to find the differences between T2DM patients with and without depressive symptoms. Significant differences were found in the rates of myocardial infarction and stress, work status, sleeping hours, educational background, average salary, and in BMI and in levels of TC, TG, urea, creatinine, uric acid, and homocysteine. Notably, educational level and average salary were higher in the depressive symptoms group than in the non-depressive symptoms group. It may be possible that the patients in the depressive symptoms group had more pressures at their work.

Among the factors mentioned above, it was found that amount of sleeping hours, history of myocardial infarction and stress in one month, working status, and TC were significantly associated with the occurrence of depressive symptoms in T2DM patients by logistic regression. Thus, in future studies, researchers should pay more attention to patients who comply with the factors mentioned above, and intervene on behalf of diabetic patients with regard to those factors that play a significant role in the prevention of depressive symptoms.

This study was conducted at the community level and without the influence of the hospital environment on diabetic patients, thus selection bias was reduced. Furthermore, this study shows for the first time the prevalence of depressive symptoms of T2DM patients in the biggest city in China. In addition, we also found statistically significant factors associated with depressive symptoms.

Although this study produced important results, there were some limitations. First, the diagnosis of depressive symptoms was by ZSDS only. It would have been more reliable if the study diagnosed depressive symptoms combined with other scales. Specifically, the average age of the participants in the study was 70.13 years and for people of this age, diagnosis depressive symptoms combined with the Geriatric Depression Scale may be more appropriate. Second, the patients in this study were from one community only, which may not reflect the total disease status of Shanghai. Third, it was not possible to control for other potentially significant unmeasured factors such as family history of other diseases, or other environmental factors, or therapeutic adherence. Combined with the findings of previous studies that both micro- and macro-vascular diabetic complications are enhanced by the presence of depression [19] in T2DM patients, and that depression may speed the death of diabetic patients [20,21], future studies will be necessary to investigate more patients from different communities, and more factors will need to be measured that may be associated with depressive symptoms in Shanghai or the entirety of China. Bogner et al [22] also found that if older individuals with depression and diabetes were treated in a timely manner, they were less likely to die within a 5-year interval. Thus, therapeutic intervention of diabetic patients with depressive symptoms is needed as early as possible.

## Conclusions

This study found that about 35.1% of patients with T2DM in Shanghai experience depressive symptoms. It also revealed that sleeping hours, history of myocardial infarction and stress in one month, working status, and TC were significantly associated with the occurrence of depressive symptoms. Because it has been shown that depression is under-recognized and undertreated [21], and depressive symptoms may negatively influence the long-term outcome of diabetic patients [23], it would be crucial to screen depressive symptoms in diabetic patients and intervene with effective treatment methods as early as possible.

## Abbreviations

ZSDS: Zung Self-rating Depression Scale
T2DM: Type 2 diabetes mellitus
BMI: Body mass index
TC: Total cholesterol
TG: Triglyceride
FBG: Fasting blood glucose
SBP: Systolic blood pressure
DBP: Diastolic blood pressure
CI: confidence interval
